# Carbohydrate Estimation by a Mobile Phone-Based System Versus Self-Estimations of Individuals With Type 1 Diabetes Mellitus: A Comparative Study

**DOI:** 10.2196/jmir.5567

**Published:** 2016-05-11

**Authors:** Daniel Rhyner, Hannah Loher, Joachim Dehais, Marios Anthimopoulos, Sergey Shevchik, Ransford Henry Botwey, David Duke, Christoph Stettler, Peter Diem, Stavroula Mougiakakou

**Affiliations:** ^1^ ARTORG Center for Biomedical Engineering Research University of Bern Bern Switzerland; ^2^ Division of Endocrinology, Diabetes and Clinical Nutrition Bern University Hospital – Inselspital Bern Switzerland; ^3^ Department of Emergency Medicine Bern University Hospital – Inselspital Bern Switzerland; ^4^ Roche Diabetes Care, Inc Indianapolis, IN United States

**Keywords:** diabetes mellitus, type 1, carbohydrate counting, computer vision systems, food recognition, meal assessment, mobile phone, food volume estimation

## Abstract

**Background:**

Diabetes mellitus is spreading throughout the world and diabetic individuals have been shown to often assess their food intake inaccurately; therefore, it is a matter of urgency to develop automated diet assessment tools. The recent availability of mobile phones with enhanced capabilities, together with the advances in computer vision, have permitted the development of image analysis apps for the automated assessment of meals. GoCARB is a mobile phone-based system designed to support individuals with type 1 diabetes during daily carbohydrate estimation. In a typical scenario, the user places a reference card next to the dish and acquires two images using a mobile phone. A series of computer vision modules detect the plate and automatically segment and recognize the different food items, while their 3D shape is reconstructed. Finally, the carbohydrate content is calculated by combining the volume of each food item with the nutritional information provided by the USDA Nutrient Database for Standard Reference.

**Objective:**

The main objective of this study is to assess the accuracy of the GoCARB prototype when used by individuals with type 1 diabetes and to compare it to their own performance in carbohydrate counting. In addition, the user experience and usability of the system is evaluated by questionnaires.

**Methods:**

The study was conducted at the Bern University Hospital, “Inselspital” (Bern, Switzerland) and involved 19 adult volunteers with type 1 diabetes, each participating once. Each study day, a total of six meals of broad diversity were taken from the hospital’s restaurant and presented to the participants. The food items were weighed on a standard balance and the true amount of carbohydrate was calculated from the USDA nutrient database. Participants were asked to count the carbohydrate content of each meal independently and then by using GoCARB. At the end of each session, a questionnaire was completed to assess the user’s experience with GoCARB.

**Results:**

The mean absolute error was 27.89 (SD 38.20) grams of carbohydrate for the estimation of participants, whereas the corresponding value for the GoCARB system was 12.28 (SD 9.56) grams of carbohydrate, which was a significantly better performance ( *P*=.001). In 75.4% (86/114) of the meals, the GoCARB automatic segmentation was successful and 85.1% (291/342) of individual food items were successfully recognized. Most participants found GoCARB easy to use.

**Conclusions:**

This study indicates that the system is able to estimate, on average, the carbohydrate content of meals with higher accuracy than individuals with type 1 diabetes can. The participants thought the app was useful and easy to use. GoCARB seems to be a well-accepted supportive mHealth tool for the assessment of served-on-a-plate meals.

## Introduction

The disease burden related to diabetes mellitus (hereafter, diabetes) is high and is still rising globally, fuelled by the global rise in the prevalence of obesity and unhealthy lifestyles. The latest estimates show a global prevalence of 387 million people with diabetes in 2015, which is expected to rise to 592 million by 2035 [1]. Of every US $9 spent in health care, US $1 is spent on diabetes corresponding to an estimated expenditure of US $612 billion worldwide in 2014 [2]. Premature morbidity, mortality, reduced life expectancy, financial, and other costs of diabetes make it one of the key public health conditions of the 21st century. Type 1 and type 2 diabetes are the two main types, with type 2 diabetes accounting for the majority (>85%) of total diabetes prevalence [1,3]. Approximately 5% to 15% of patients suffer from type 1 diabetes. Type 1 diabetes is an autoimmune process that completely destroys the insulin-producing pancreatic beta cells, leaving the individuals dependent on exogenous insulin. Achieving a metabolic state close to the physiological is a very challenging task and involves glucose monitoring, insulin treatment, diet management, controlled physical activity, and continuous education on diabetes management. Despite the availability of new drugs, advanced educational programs, and technical solutions that permit continuous glucose monitoring and subcutaneous insulin infusion (insulin pumps), episodes of hypo- and hyperglycemia are still common and the risk of micro- and macrovascular diseases related to diabetes (eg, cardiovascular diseases, retinopathy, and nephropathy) is high.

Estimating the amount of insulin to deliver is one of most challenging and essential tasks in the everyday life of individuals with diabetes. Counting the carbohydrate content of meals to be consumed is a cornerstone of optimal insulin dose estimation. It has been shown that an inaccuracy of ±10 grams does not impair postprandial glycemic control in children [4], but a variation of ±20 grams significantly affects postprandial glycemia [5]. To this end, individuals with diabetes attend nutritional courses on carbohydrate counting; however, according to numerous studies, patients face recurrent difficulties [6-9]. Meals on a plate are especially prone to carbohydrate underestimation, which underlines the emergent need for novel approaches to carbohydrate estimation. The debate on how to optimally estimate carbohydrate intake and facilitate advanced carbohydrate-counting regimes is ongoing and controversial [10].

Over the last two decades, a number of apps have been introduced to track carbohydrate consumption. A recent systematic review analyzed 31 digital approaches to record food intake and nutrition [11]. These systems use different devices—mostly mobile phones, followed by personal computers, and, in older studies, personal digital assistants. They are primarily designed for users who are overweight or obese, with diabetes mellitus, or who want to stay healthy. However, the vast majority of the apps reviewed, except for two using a barcode scanning function, relied on manual input of data either by typing or by selecting a food type from a database. Other proposed systems employ trained health care workers located at a remote location and giving advice on food type, volume, or calorie content. One example is the remote food photography method that relies on the user to take mobile phone pictures of her/his meals, snacks, and beverages, which are then remotely transferred to a trained person who compares the meal to an existing image database and rates its nutrient or caloric content [12]. However, these methods have many limitations in terms of user-friendliness, cost-effectiveness, availability on a large scale, and reproducibility. In a recent study [13], a mobile phone app using augmented reality was proposed that facilitates the estimation of a meal’s carbohydrate content by considering its three-dimensional (3D) shape as drawn by the user on the mobile phone screen. According to this study, the system helped users improve their carbohydrate estimation skills, although the required manual input is still burdensome.

All methods presented for nonpacked food assessment have either relied on manual user input or involve a remotely located human component. The recent advances in computer vision and the widespread use of mobile phones with enhanced capabilities have permitted the development of image analysis apps for the automatic assessment of food intake. The input of such an app is a number of images or a short video of the upcoming meal as captured by the user’s mobile phone camera. A series of image analysis steps follows, executed either on the mobile phone or on a remote server, to recognize the types and quantities of the meal’s food items and to estimate the corresponding nutritional information. Over the last few years, several systems have been proposed with different assumptions, input requirements, and algorithmic approaches [14-16].

The first attempt was made by DiaWear, a system aiming to provide calorie information to diabetic patients [17]. DiaWear considers four fast-food classes and requires one image, with the foods placed on a lighter background and separated from one another. The nutritional content is found directly from reference tables without attempting to estimate the volume. The Pittsburgh Fast-food Image Dataset was created to test food recognition algorithms and contains seven classes of fast-food products [18,19] but, again, the food portion is not considered. The system developed within the Technology-Assisted Dietary Assessment project uses one meal image and covers 19 food classes [20]. Food volume is estimated by fitting spherical or prismatic 3D models on the detected food areas. However, the use of a single image to estimate the food’s volume inevitably depends on weak assumptions about its 3D shape. The use of multiple images by some systems enhanced the results, especially for the volume estimation. The Food Intake Visual and Voice Recognizer [21] uses a short video of the meal from which three images are extracted and used for dense stereo reconstruction and volume estimation. For recognition, 26 food types are supported; nevertheless, the system relies on the user to enter food types through speech because this enhances classification accuracy. DietCam [14], a system for calorie estimation, requires three images or a video of a meal and segments/recognizes food items by visually matching them to a database. The volume is estimated by sparsely reconstructing its 3D shape and fitting an appropriate model. However, critical system characteristics, such as the food classes considered, are not specified. Pouladzadeh et al [15] proposed a system for calorie measurement considering 30 food classes and using the thumb as a reference object. The first image is taken from the top to estimate the food area, the second from the side to estimate height, and then the two values are multiplied to obtain the volume. However, capturing the dish from the side causes occlusions, while assuming constant height for all the food items will introduce large errors as does any misplacement of the thumb.

GoCARB is a novel system for carbohydrate estimation designed for individuals with type 1 diabetes and aims to achieve carbohydrate estimation with an error less than 20 grams per meal. In a previous study, the system was technically evaluated using 24 multifood dishes [16]. The entire evaluation was performed by the researchers involved under controlled conditions. The results showed that the prototype was able to estimate the carbohydrate content with a mean absolute error in the order of 10% (SD 13) or mean 6 (SD 8) carbohydrate grams per meal. The scope of the present study is to assess the performance of the GoCARB prototype when used by individuals with type 1 diabetes and compare it to their own performance in carbohydrate counting.

## Methods

### GoCARB System

**Figure 1:**
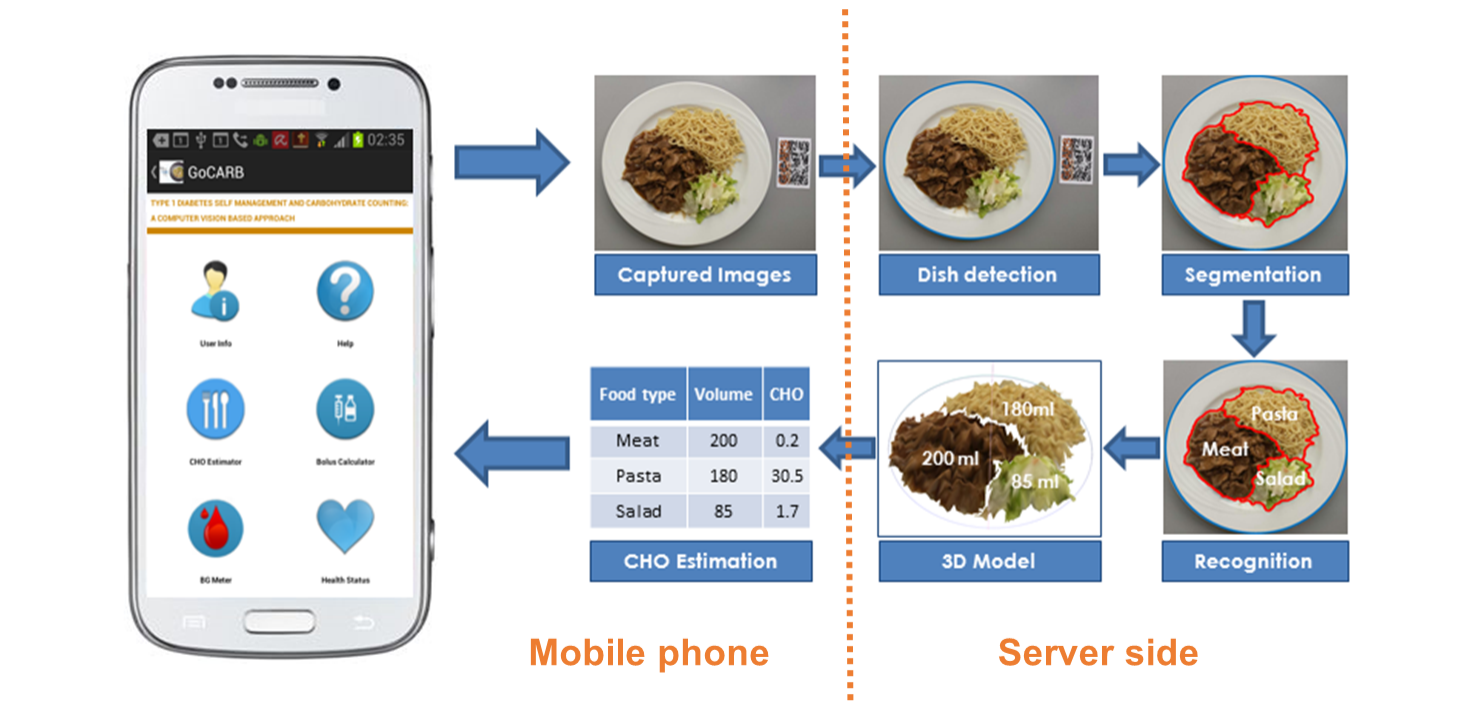
Overview of the GoCARB system.

**Figure 2:**
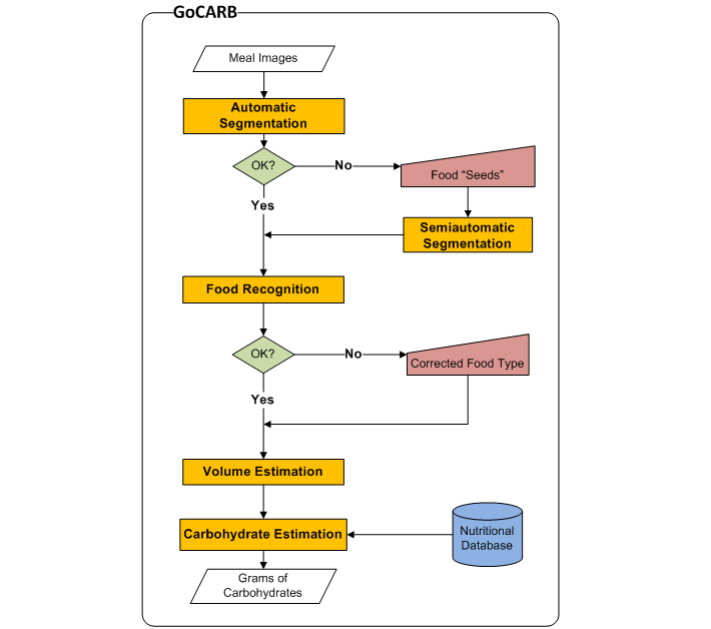
Flowchart of the GoCARB system.

**Figure 3:**
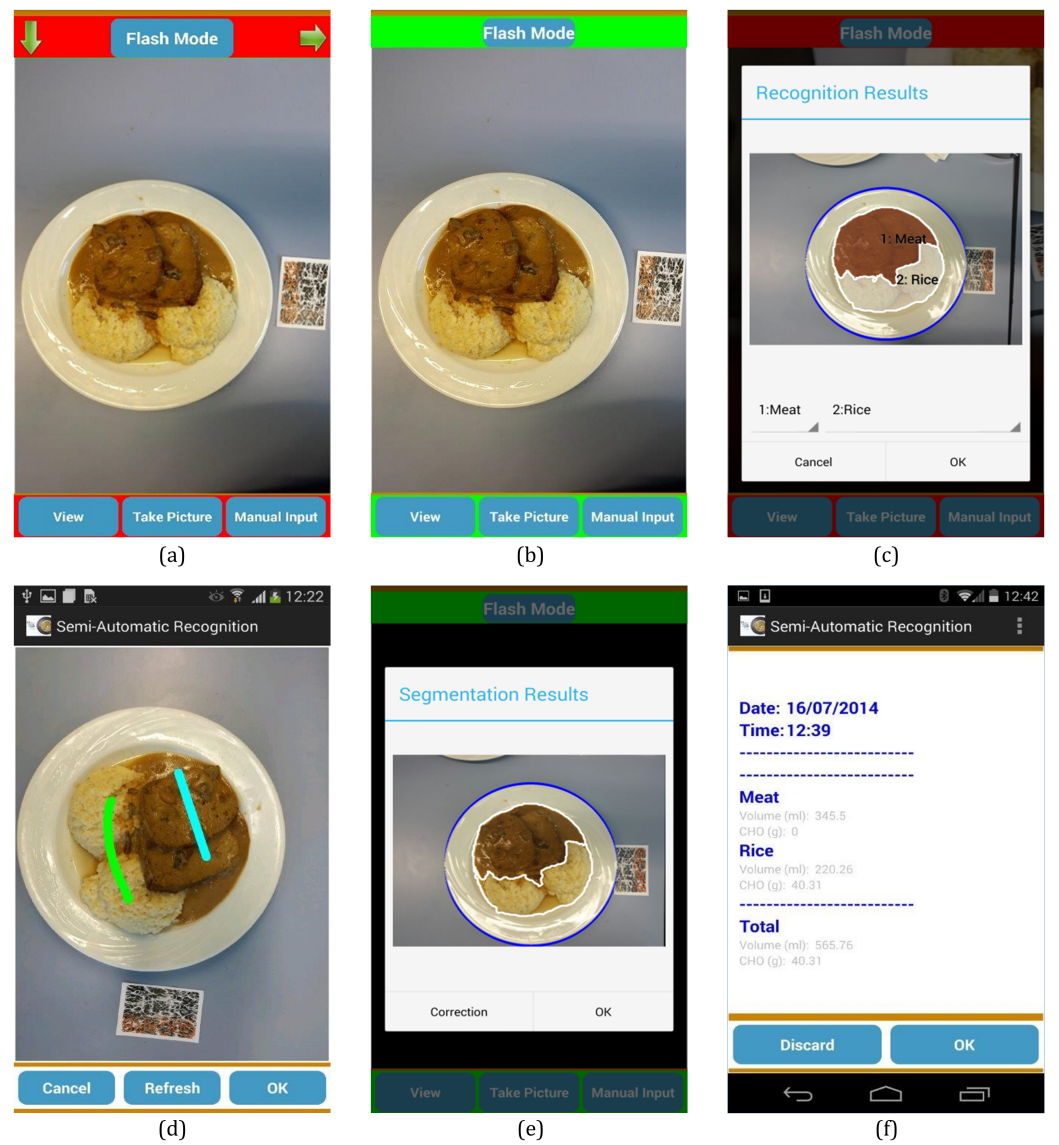
Screenshots of the GoCARB app: (a) the red frame indicates wrong angle so image acquisition is disabled, (b) when the frame turns green, the user can take an image, (c) the result of the automatic segmentation, (d) user-given seeds required for the semiautomatic segmentation, (e) the result of the recognition with the option of manual correction, and (f) the results screen displayed to the user.

GoCARB is a novel system that aims to support individuals with type 1 diabetes in carbohydrate counting. The system runs on Android mobile phones and uses computer vision to estimate the carbohydrate content of meals. An overview of the system’s main intermediate results is presented in Figure 1, whereas the corresponding flowchart is shown in Figure 2. For each estimation, the user places a reference card next to the meal and acquires two images using the camera of the mobile phone. The images are acquired at 0±3 degrees and at 15±3 degrees from the vertical axis crossing the center of the dish, respectively. A graphical user interface supports the user in choosing the optimal angles for each image based on the built-in motion sensors of the mobile phone; the user is only allowed to take the picture when the frame turns green (Figure 3 a-b). The system was designed to have minimum assumptions; namely, that the scene contains only one dish, which should be round, and that there should be no occlusion among the different food items in the dish. After acquisition, the images are transmitted to a dedicated server via Wi-Fi or the mobile network, where a series of computer vision operations are performed. All computer vision modules run on the server, whereas the mobile phone is used for image acquisition, calculation of carbohydrate values, and visualization of the results.

The first step in the series is to detect the dish and automatically segment the different food items in it [22] (Figure 3 c). The dish is detected by extracting the edges of the image and applying a robust fitting paradigm to find an elliptical plate border. The accuracy of this step was estimated to be over 99%, which is essential because the rest of the system relies on it. Automatic segmentation then grows homogenous color regions on a grid inside the dish and merges them on the basis of their color distance and mutual edge size until a minimum size is reached. The accuracy of this module is over 88% and, if it fails, an interactive segmentation tool can be used, which is reliable even for the most difficult cases. To this end, the user has to roughly indicate the position of each food item by touching the screen of the phone (Figure 3 d). These user-given points are used as “seeds” instead of a grid to grow homogenous color regions that correspond to each food item. As soon as the segmentation result is approved by the user, automatic recognition [23] is applied to each segmented food item by using color and texture features fed to a support vector machine. Nine broad food classes are considered; namely, pasta, potatoes, meat, breaded (eg, schnitzel), rice, green salad/vegetables, mashed potatoes, carrots, and beans. The accuracy of this automatic recognition is over 85%. If the result is wrong, the user may correct the system by choosing the right food class from an ordered list in accordance with the confidence of the classifier (Figure 3 e). The food’s 3D shape is then reconstructed [24], utilizing both acquired images and the reference card. Key points are detected and matched between the two images to define the orientation and location of the images in space. Using this information, all image pixels are put in correspondence between the two images and their disparity provides the depth used to build the 3D model. By using the 3D model and the segmentation results, the volume of each item is calculated and used to obtain the corresponding carbohydrate content based on the US Department of Agriculture (USDA) nutrient database [25]. For each of the nine food classes, all relevant entries in the database are identified and the mean carbohydrate density is assigned to it. Finally, the results are transmitted back to the mobile phone and displayed to the user (Figure 3 f). More detailed information on technical specifications can be found elsewhere [16,22-24].

### Study Setup

**Figure 4:**
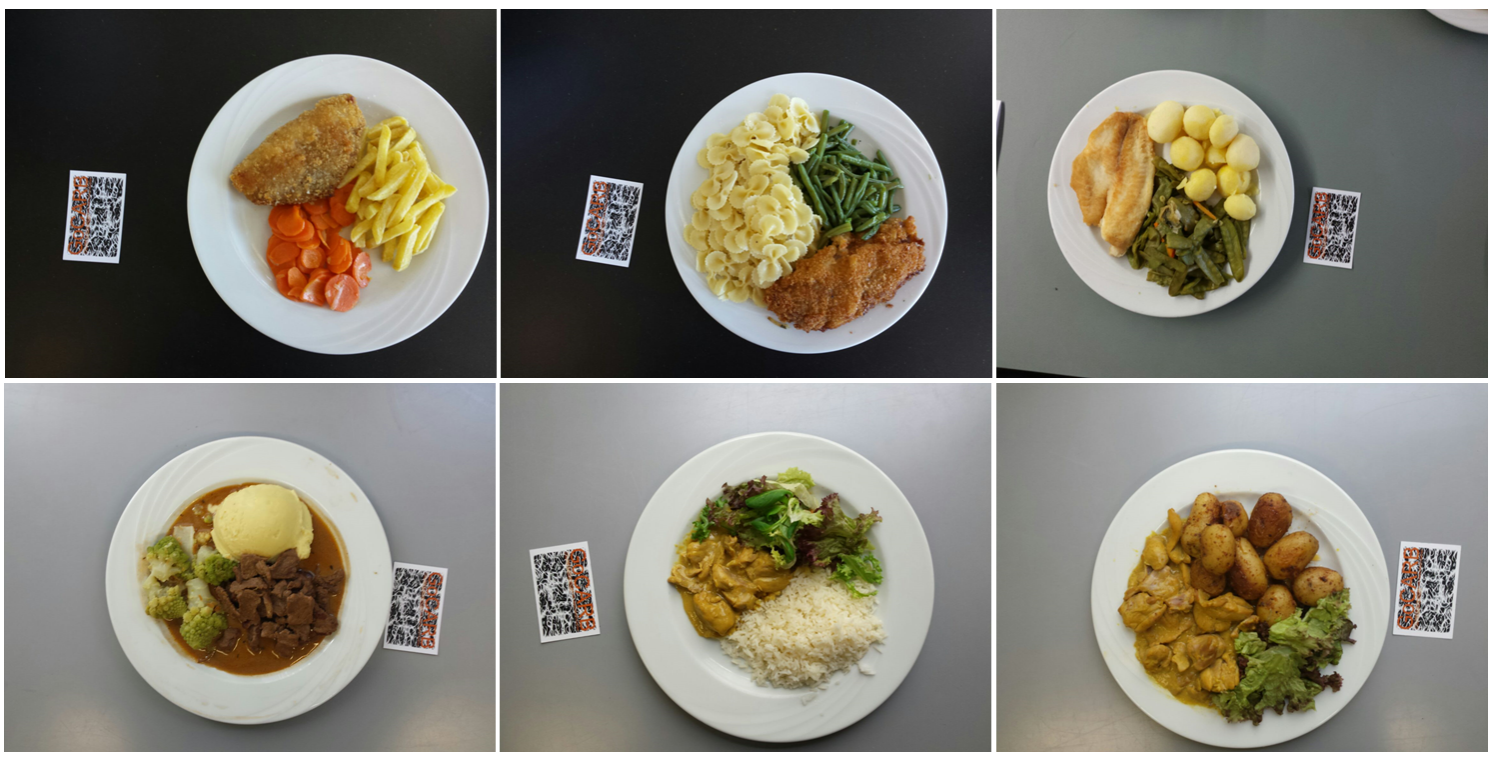
Examples of the dishes used for the study.

Nineteen adult individuals receiving regular care at the Division of Endocrinology, Diabetes and Clinical Nutrition of the Bern University Hospital, “Inselspital” (Bern, Switzerland) were asked to participate in the GoCARB study, which lasted a total of 10 days during July and August 2014. The GoCARB system was preinstalled on two types of mobile phones, Samsung Galaxy S4 and Nexus 5, both running the Android operation system (version Jelly Bean 4.2). Every day of the study, two standard dishes were ordered from the hospital restaurant, each in three sizes (small, normal, and large), resulting in six different dishes per day and 60 dishes in total. Each dish contained three food items that corresponded to common sources of protein (eg, meat, fish), carbohydrates (eg, pasta, rice), and vegetables/salads (eg, lettuce, carrots). Figure 4 presents some examples of these dishes. The food items were weighed with a household scale and the true carbohydrate content in grams was defined using the USDA National Nutrient Database for Standard Reference. Each participant joined the study for one session during which she/he was asked to estimate the carbohydrate content of each of the six meals on her/his own and then by using the GoCARB system. The 19 participants were randomly distributed over the 10 days of the study so some dishes were shown to multiple participants. Therefore, a total of 114 (19×6) estimations were made even though there were 60 unique dishes. Before using the app, every participant received short training and a detailed written user manual. At the end of each session, a questionnaire was completed to assess the user’s experience with GoCARB. The questionnaire was a combination of closed and open questions and was used to gather information about the satisfaction and perceived usefulness of the mobile phone app. Because this study did not collect nor analyze patient-specific clinical data, the study was exempted from formal ethical approval.

### Participants

The mean age of the participants was 40.5 (SD 11.5) years and all but one were mobile phone users. Among the mobile phone owners, 12 were familiar with iOS, five with Android, and one with Blackberry OS. Of these, 16% (3/19) used a nutrition-related app in their everyday life, whereas only 11% (2/19) used a health-related app.

### Statistical Analysis

For statistical comparison, we used the mean absolute error of the participants’ estimates with and without using GoCARB. The data were nonnormally distributed; therefore, an independent Mann-Whitney *U*test was applied for significance testing. Descriptive statistical analysis was performed in open-source software R [26] and SPSS version 23 (IBM Corp, Armonk, NY, USA). The questionnaire investigating usability and user satisfaction included 24 Likert-scale questions, three polar questions, and three open questions. Likert-scale questions ranged from “do not agree at all,” “do not agree,” “neutral,” “agree,” to “agree strongly” for general GoCARB usability and from “very bad,” “bad,” “neutral,” “good,” to “very good” for the performance of automatic and interactive GoCARB features.

## Results

### Carbohydrate Estimation: GoCARB Versus Individuals With Type 1 Diabetes

As presented in Table 1, the mean absolute error of the participants with type 1 diabetes was 27.89 (SD 38.20) grams of carbohydrate, whereas the corresponding values for the GoCARB system was 12.28 (SD 9.56) grams of carbohydrate, less than the initial target of 20 grams. This improvement in the estimation error was statistically significant ( *P*=.001). The corresponding mean relative error in carbohydrate estimation was 54.8% (SD 72.3%) for the participants and 26.2% (SD 18.7%) for GoCARB.

**Table 1:** Performance of participants (N=19) in carbohydrate estimation with and without GoCARB.

All participants	Absolute error (grams), mean (SD)	Absolute percentage error (%), mean (SD)	Absolute errors <20 grams, n (%)
Without GoCARB	27.89 (38.20)	54.8 (72.3)	67/114 (58.8)
With GoCARB	12.28 (9.56)	26.2 (18.7)	92/114 (80.7)

### Distribution of Estimation Errors

**Figure 5:**
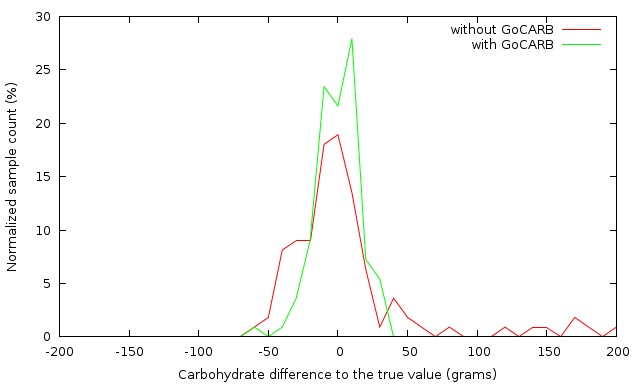
Distribution of the absolute errors in carbohydrate estimation with and without GoCARB.

**Figure 6:**
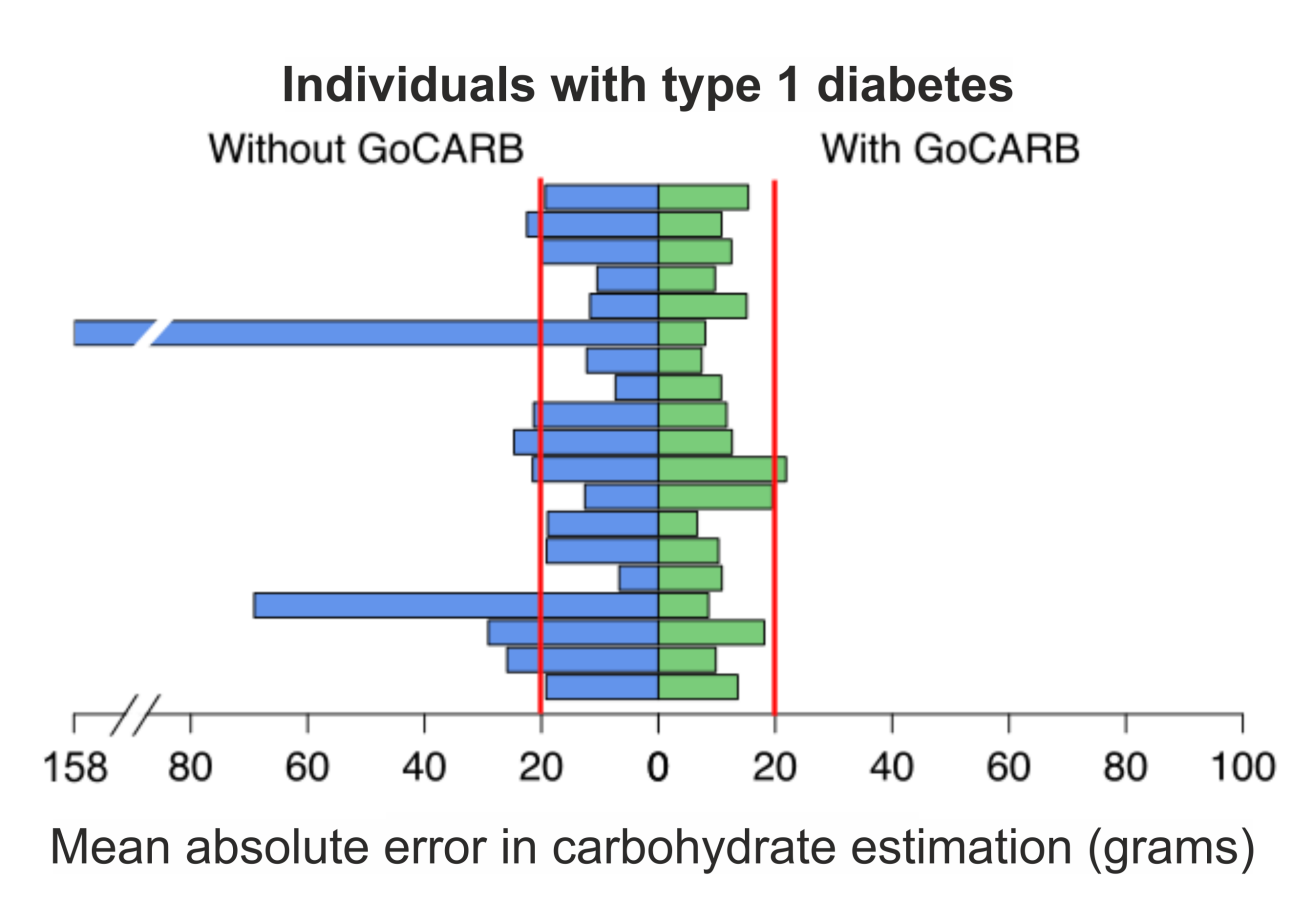
Distribution of the mean absolute error per participant with and without GoCARB. The vertical lines represent the target range of errors.

Figure 5 provides the distribution of errors for carbohydrates as counted by the individuals with type 1 diabetes and as estimated by using the GoCARB system. In the case of self-assessment, the error distribution was broad with outliers up to 200 grams of carbohydrate. In the case of GoCARB, the errors were symmetric and concentrated around zero. In general, the individuals with type 1 diabetes more frequently (60.5%, 69/114) underestimated the carbohydrates of a meal; however, the error was higher in the case of the overestimation. The GoCARB system exhibited an evenly balanced distribution of under- and overestimation, 50.9% (58/114) and 49.1% (56/114), respectively, whereas the errors were almost of the same magnitude for both under- and overestimations. Furthermore, in 58.8% (67/114) of the cases of carbohydrates counted by the individuals with type 1 diabetes, the error was in the range of –20 grams to +20 grams, whereas with GoCARB the estimations were in the required range in 80.7% (92/114) of the cases. The analysis was followed by looking for outliers in carbohydrate estimations as counted by the individuals with type 1 diabetes. Examining the data (see Figure 6), one participant was identified who consistently overestimated all her/his meals. The mean absolute estimation error for this participant was 158.19 (SD 26.09) grams of carbohydrate. As seen in Table 2, even by excluding the one participant with extreme carbohydrate values, the use of GoCARB resulted in significantly better performances ( *P*=.01).

**Table 2:** Performance of participants in carbohydrate estimation with and without GoCARB after excluding one participant with extreme errors (n=18).

Excluding extremely bad estimator	Absolute error (grams), mean (SD)	Absolute percentage error (%), mean (SD)	Absolute errors <20 grams, n (%)
Without GoCARB	17.81 (14.94)	34.3 (24.3)	67/108 (62.0)
With GoCARB	12.75 (9.84)	26.9 (18.9)	86/108 (79.6)

### Influence of Meal Size on Accuracy

For each meal, three different meal sizes were presented to the user. With larger meals, individual’s counting errors also increased: mean 18.47 (SD 28.86) grams of carbohydrate for small meals, mean 26.39 (SD 38.20) grams of carbohydrate for medium meals, and mean 38.82 (SD 47.03) grams of carbohydrate for large meals. When the participants used the GoCARB system to estimate the carbohydrate content of the meals, the error exhibited less variation across the different meal sizes: mean 10.34 (SD 6.16) grams of carbohydrate for small, mean 10.12 (SD 8.02) grams of carbohydrate for medium, and mean 16.38 (SD 12.30) grams of carbohydrate for large-sized meals.

### Usage of Interactive Features

In the GoCARB prototype, the user is able to interact with the system when she/he is not satisfied with the fully automatic segmentation and/or recognition results. Thus, the system can be corrected, while the volume estimation is completed. During the study, the automatic segmentation was successful in 75.4% (86/114) of the cases. For recognition, the system achieved correct recognition of all three distinct food items on a dish in 59.6% (68/114) of cases. For these cases, manual correction was not needed. In 36.0% (41/114) of cases, the system was able to recognize two of three food items correctly, whereas only one food item was recognized in 4.4% (5/114) of cases. For these cases, the user had to manually correct the system. In all cases, GoCARB recognized correctly at least one of the food items. Out of a total of 342 food items in 114 meals, 85.1% (291/342) were correctly automatically recognized.

### User Experience

**Figure 7:**
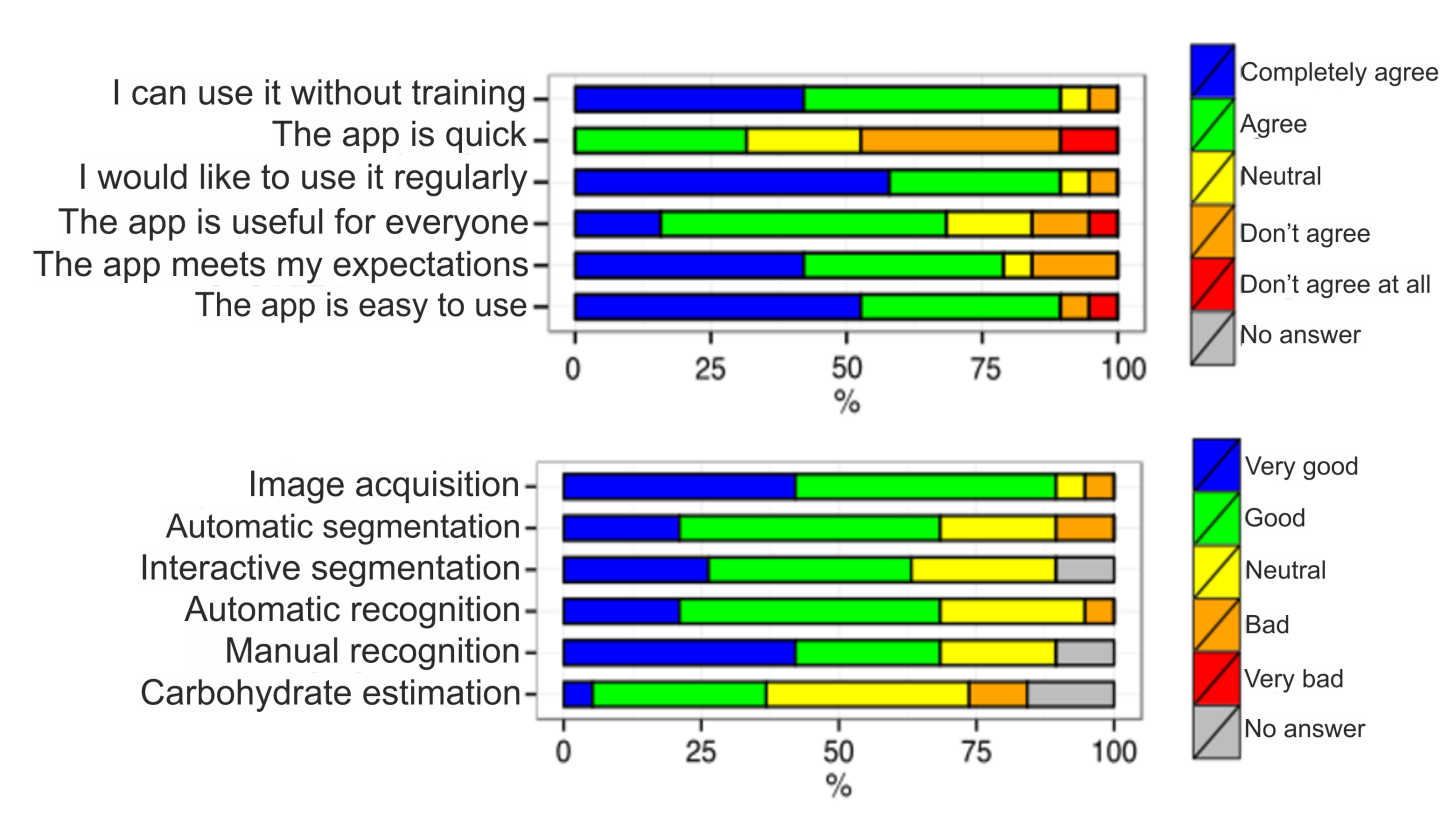
Graphical representation of the participants' answers to the questionnaire.

To evaluate the user perception and usability aspects of GoCARB, a questionnaire was given to participants. The majority (90%, 17/19) agreed or agreed completely that GoCARB was easy to use (Figure 7). Furthermore, 90% (17/19) of participants would have liked to use GoCARB on a regular basis and 68% (13/19) thought GoCARB was useful for all individuals with type 1 diabetes. The processing speed of the GoCARB system was considered too slow by 47% (9/19). It is noteworthy that the client-server architecture of the system made its speed highly dependent on 3/4G or Wi-Fi signal strength. The GoCARB workflow was mostly rated favorable, with moderate to high approval of automated and interactive steps. The feedback from the open questions was similar. The participants were asked to list negative and positive aspects of the app and possibly to provide suggestions for improving the system. Most of the negative feedback was related to processing delays and the dependence on the Internet connection. As for positive aspects, the participants indicated the ease of use and the usefulness of such an app. Many participants proposed that the system should be extended to other food types, such as desserts.

## Discussion

### Principal Findings

The results of the study indicate that GoCARB is, on average, more accurate at estimating carbohydrate content than the individuals with type 1 diabetes who participated. If all participants are included, the mean absolute estimation error while using GoCARB (12.28 grams of carbohydrate) was reduced by more than 50% than without using GoCARB (27.89 grams of carbohydrate). If it is considered that an absolute error of 20 grams or more gives a significant increase in risk of hyper- or hypoglycemia, GoCARB fulfilled this prerequisite for 80.7% (92/114) of the cases. Moreover, the relatively low standard deviation (SD 9.56 grams) of GoCARB’s errors, along with their symmetric distribution around zero, demonstrates the relative stability and consistency of the system. In 75.4% (86/114) of the meals, GoCARB’s automatic segmentation was successful and 85.1% (291/342) of individual food items were successfully recognized. With regard to user satisfaction, the majority of participants found the system easy to use and expressed strong interest in using it on a daily basis. Among these individuals with type 1 diabetes, there was a strong consensus that an app such as GoCARB could benefit everybody with diabetes. The only concern of the participants was related to the speed of the app (approximately 1 min including user interactions with the system), which was mainly due to delays in the transmission of data over an unstable wireless network.

The errors in carbohydrate estimation by the participants exhibited a wide spread, but only one participant was responsible for all the estimates beyond the 95th percentile. Despite different approaches to outlier exclusion, the overall difference between individuals with type 1 diabetes with and without GoCARB remains significant. Whether it is reasonable to include outliers in our statistical analysis greatly depends on the point of view. Although statistical analysis permits outlier exclusion, clinical reasoning and experience supports their inclusion, especially because everyday clinical life shows that extremes are possible and might even be the accidental starting point of glycemic decompensation, in extremis leading to hospitalization or death. Undoubtedly, these diabetics with weak estimation skills would benefit the most. Nevertheless, even after excluding this participant, the difference in accuracy between the conventional methods and GoCARB remains statistically significant ( *P*=.01).

### Comparison With Previous Work

There have been several systems proposed using single/multiple food images or videos to recognize food items and calculate the corresponding volumes [14-21]. However, to the best of our knowledge, there has been no comparable study evaluating an automatic dietary assessment system together with end users. From our experience with the GoCARB system, we believe that a thorough scientific evaluation and an appropriate framework for mobile medical app development and evaluation are of the utmost importance. Only a few commercially available medical apps undergo in-depth scientific evaluation and there is virtually no support provided to health care professionals or patients to help them to select the most beneficial app. This concern is increasingly being addressed and steps have been taken to develop guidelines and standards [27,28].

### Limitations

This is a preclinical study and the sample number was chosen for reasons of convenience. Within the study sample, there was a wide spread of estimation error and estimation error was significantly higher than that reported in the literature. Nevertheless, we believe that this is concordant with many patient groups found in real life, but only studies with increased sample size may bring certainty. Moreover, we do not know if or when participants last had a nutritionist-guided training session on carbohydrate counting. Because there is general agreement that regular teaching and training significantly improve estimation capability [29], such information is of interest, especially to identify individuals with type 1 diabetes who perform poorly despite adequate training.

Furthermore, purely automatic computer vision-based systems have certain limitations. For complex meal types with multiple ingredients mixed arbitrarily or meals covered by sauce, additional information is needed by the user on the food type (eg, lasagne). The rest of the modules are independent of food type. Macronutrients, such as fat, are nearly impossible to quantify by computer vision only, but can still affect a person’s postprandial glycemia. Moreover, the type of carbohydrate also affects the postprandial response, apart from its amount. Intrinsic variables that influence the effect of carbohydrate-containing meals on blood glucose response include the specific type of food ingested, type of starch (amylose vs amylopectin), style of preparation (cooking method and time, amount of heat or moisture used), ripeness, and degree of processing. To some extent, this is reflected in the glycemic index of foods and is an established method to compare the physiological postprandial glucose responses to different types of carbohydrate-containing foods [30]. Essential components of a system such as GoCARB are the multimedia and nutritional databases used for recognizing the food and calculating its nutritional profile. Generating and expanding a food image dataset to provide a broad variety for automatic food recognition is a challenging and often costly task. In addition, although technology has improved the accessibility of nutritional databases, the availability of up-to-date food composition data for many food items is still limited. Although new products continually appear in the food supply, gaps will always exist between what databases contain and what individuals consume.

### Future Research

We plan to optimize the existing prototype and expand its functionalities. The code of the system will be optimized and components will be moved from the server to the mobile phone side to increase its speed and efficiency. The food types considered will be expanded to cover a wider spectrum of cultures and eating habits. Integration of a barcode reader would be easily feasible technically and would allow us to cover packaged foods. Other input methods are promising; these might include voice input or dropdown lists specifying details of a meal that cannot be assessed by a picture (eg, fried vs boiled, cooked vs uncooked, olive oil vs sunflower oil).

Although GoCARB aims to support individuals with type 1 diabetes, the system might be extended so that it not only displays carbohydrates, but also other micro- and macronutrients. The system could then be used to facilitate diet management. Such a system could be useful for a large heterogeneous group of medical conditions related to food, food intake, and digestion or weight management. It could also be aimed toward a more general and healthy population in an attempt to encourage people to make more deliberate food choices leading to a healthier lifestyle. Such a tool would not only be of interest to individuals, but also to the nutrition research community because conventional methods of diet assessment, such as the 24-hour food recall method, food intake questionnaires, or a paperback food diary, are difficult to apply, are time consuming, and are known to be consistently inaccurate.

### Conclusion

In this study, we have presented the evaluation of a novel mobile phone-based system to estimate the carbohydrate content of a meal on a plate using computer vision. The GoCARB system proves to be a reliable support tool for carbohydrate estimation of meals on a plate and provides more accurate carbohydrate estimates than those of our cohort of participants with type 1 diabetes using conventional methods. Among the participants, there was a strong consensus that an app such as the GoCARB system could benefit everybody with diabetes. We believe that computer vision has the potential to facilitate and ameliorate the cumbersome and error-prone task of carbohydrate estimation. The output of GoCARB could be used as input to a bolus calculator that will also consider the personal characteristics of the user (eg, insulin-to-carbohydrate ratio) and suggest an insulin dose. After further development, GoCARB could ultimately make a distinct contribution to a fully automated artificial pancreas.

## Abbreviations

USDA: US Department of Agriculture
